# Sulforaphane substantially impedes testicular ferroptosis in adult rats exposed to di-2-ethylhexyl phthalate through activation of NRF-2/SLC7A11/GPX-4 trajectory

**DOI:** 10.1007/s00210-024-03440-w

**Published:** 2024-10-01

**Authors:** Mohammed M. Elseweidy, Nouran G. Harb, Abdelmoniem A. Ali, Reda M. Abd El-Aziz, Rania A. Elrashidy

**Affiliations:** 1 Department of Biochemistry, Faculty of Pharmacy, Zagazig University, Zagazig, 44519 Egypt; 2 Department of Pathology, Faculty of Veterinary Medicine, Zagazig University, Zagazig, 44519 Egypt; 3 Department of Physiology, Faculty of Veterinary Medicine, Zagazig University, Zagazig, 44519 Egypt

**Keywords:** Ferroptosis, Sulforaphane, Di-2-ethylhexyl phthalate, Testes, ACSL4, NRF-2

## Abstract

Di-2-ethylhexyl phthalate (DEHP) is a common plasticizer with a deleterious impact on testicular functionality and male fertility. Growing evidence implicates ferroptosis as one of the plausible mechanisms for DEHP-induced testicular injury. Sulforaphane (SFN) is a natural isothiocyanate displaying beneficial effects on testicular injury in several animal models. Herein, we explored the potential protective effect of SFN on testicular ferroptosis and toxicity evoked by DEHP. Adult male Wistar rats were equally distributed into three groups (*n* = 6/group): (i) CON group; (ii) DEHP group, received DEHP (2 g/kg PO) for 4 weeks; and (iii) DEHP + SFN group, received SFN (10 mg/kg, PO) 1 week prior to DEHP then concurrently with DEHP for further 4 weeks. Compared to CON group, exposure to DEHP caused testicular atrophy, deteriorated testicular architecture, testicular fibrosis, reduced sperm count and motility, higher sperm deformity, and declined serum testosterone level. All these abnormalities were ameliorated by SFN preconditioning. Additionally, pretreatment with SFN reversed the increased aromatase level and upregulated the steroidogenic markers in testes of DEHP-exposed rats. SFN pretreatment also counteracted DEHP-induced oxidative stress and boosted the total antioxidant capacity in testicular tissue via activation of the nuclear factor erythroid 2-related factor 2 (NRF-2) and its downstream target, hemeoxygenase-1 (HO-1). Moreover, SFN preconditioning mitigated DEHP-induced ferroptosis through up-surging SLC7A11, GPX-4, and GSH, while suppressing iron overload and ACSL4-induced lipid peroxidation in testicular tissue of rats. These findings may nominate SFN as a promising protective intervention to alleviate testicular ferroptosis associated with DEHP exposure through activation of NRF-2/SLC7A11/GPX-4 trajectory.

## Introduction

Exposure to environmental pollutants can deteriorate male fertility. These pollutants act as endocrine disruptor chemicals (EDCs) which can disrupt the male reproductive system through either their estrogenic or anti-androgenic action (Mathur and D'cruz 2011). EDCs have various pathophysiological effects on the male reproductive system. Numerous studies have unveiled that exposure to EDCs can lead to failure of spermatogenesis, impaired sperm parameters, decreased testosterone levels, undescended testicles, or testicular cancer (Sharma et al. 2020; Thacharodi et al. 2023). There is a substantial evidence suggesting that phthalates are potent EDCs and have a negative impact on male reproductive system of humans (Chen et al. 2014).

Di-2-ethylhexyl phthalate (DEHP) is a well-known plasticizer incorporated in plastic industry. People can be subjected to DEHP via food digestion, inhalation, dermal contact, or through medical devices (Rowdhwal and Chen 2018). Studies have indicated that the typical exposure of general population to DEHP ranges between 3 and 30 µg/kg/day. Higher exposure may occur in some special medical conditions. DEHP exposure can reach much higher levels, from 1.5 mg/kg/day during hemodialysis to between 10 and 20 mg/kg/day through neonatal transfusion or parenteral nutrition (Culty et al. 2008). The 50% lethal dose (LD50) value of DEHP has been found to be ranged from 14.2 to 50 g/kg, as determined via acute oral toxicity testing in rats (Krauskopf 1973). The no observed toxic or adverse effect (NOAEL) value of DEHP has been determined to be 28.9 mg/kg, while the lowest dose at which there is an observed toxic or adverse effect (LOAEL) value of DEHP is 146.6 mg/kg, for potential testicular toxicity and carcinogenicity in rodent studies (Kim 2016). Therefore, DEHP has been classified as a carcinogenic 2B substance by the International Agency for Research on Cancer (IARC). Many pre-clinical studies have linked DEHP to multiple organ toxicities including nephropathy (Ashari et al. 2022), cardiomyopathy (Posnack 2014), and endometriosis (Kim et al. 2015). DEHP can significantly worsen the male reproductive system by decreasing testosterone synthesis, increasing germ cells death and impairing sperm functions (Hong et al. 2021). DEHP can induce testicular toxicity by impairing redox homeostasis in the testis (Zhang et al. 2022), but the precise mechanisms are not fully explored.

Ferroptosis is a distinct form of regulated cell death with prominent hallmarks including iron overload, lipid peroxidation, and glutathione (GSH) depletion. The system X_C_^−^ is a membrane transporter with two subunits (namely SLC3A2 and SLC7A11) and responsible for GSH biosynthesis. GSH is essential for the proper function of glutathione peroxidase-4 (GPX-4), which rescues the cells against lipid peroxidation and ferroptosis (Chen et al. 2021). Iron is one of the trace elements essential for the synthesis of nucleic acids and mitochondrial biogenesis, thereby playing a key role in spermatogenesis, sperm motility, steroid production, and male fertility (Tvrda et al. 2015). The importance of iron in male fertility has been evidenced from males with iron deficiency anemia who experience poor semen parameters (Akhter et al. 2021). Moreover, infertility is a well-known complication in male patients suffering from sickle cell anemia (Osegbe and Akinyanju 1987) and beta thalassemia (De Sanctis et al. 2017). Such diseases are usually accompanied by low ferritin levels and iron deficiency. On the contrary, accumulation of iron has deleterious consequences on testicular homeostasis and male fertility (Gabrielsen et al. 2018). Iron overload can enhance the oxidative stress in the testes causing a depletion of endogenous antioxidants, accompanied by oxidative damage to lipids, proteins, and DNA, impaired spermatogenesis with subsequent infertility (Mojica-Villegas et al. 2014; Wellejus et al. 2000). Excessive iron has been shown to induce testicular atrophy and lesions along with improper reproductive performance (Merker et al. 1996). Hemochromatosis is associated with hypogonadism, morphological changes in the seminiferous tubules, and abnormal sperm motility (Gottschalk et al. 2000; Gunel-Ozcan et al. 2009). Since iron overload is a crucial hallmark of ferroptosis, hence, it is not surprising that ferroptosis can be implicated in disrupted spermatogenesis, testicular injury, and impaired male fertility (Yuan et al. 2023).

Emerging evidence designates a crosstalk between DEHP exposure and ferroptosis in several cell types, including spleen (Dai et al. 2021), liver cells (Hosseinzadeh et al. 2021), cardiomyocytes (Wang et al. 2023b), renal tubular cells (Chen et al. 2023), and hippocampus neurons (Wang et al. 2023a). Importantly, an in vitro study has illustrated the involvement of ferroptosis in Leydig cell dysfunction (Guo et al. 2023). Although the pre-pubertal experience to DEHP has been demonstrated to trigger ferroptosis in testes of experimental animals (Wang et al. 2024), there is insufficient research regarding the significance of ferroptosis in DEHP-induced testicular injury in the adulthood stage. Considering the distinction between pre-pubertal and adult testes in terms of vulnerability to injury, it is essential to assess the negative effects of DEHP on adult testes.

Nuclear factor erythroid 2-related factor 2 (NRF-2) is the master intracellular defense system against redox imbalance (Wajda et al. 2016). There is an increasing evidence implicating NRF-2 as an essential pathway to maintain testicular homeostasis (Rotimi et al. 2022). This function has been evidenced from *Nrf-2* knockout male mice which exhibit enhanced testicular lipid peroxidation, germ cell apoptosis, and age-related male infertility (Nakamura et al. 2010). Hemeoxygenase-1 (HO-1) is a downstream target of NRF-2 expressed in Leydig and Sertoli cells, and exerts a key role in spermatogenesis (Shiraishi and Naito 2005). NRF-2/OH-1 can antagonize lipid peroxidation and hence may exert a potential anti-ferroptotic role (Dong et al. 2021).

Enhancement of NRF-2/HO-1 system can afford cellular defense against redox imbalance and sperm abnormalities. In this context, sulforaphane (SFN) is an isothiocyanate naturally existing in broccoli, cauliflower, and cabbage. Lately, SFN gains abundant attention owing to its powerful antioxidant capacity via promotion of NRF-2/HO-1 system (Pan et al. 2014). Preclinical studies have reported the favorable impact of SFN on testicular damage induced by dibutyl phthalate (Jiang et al. 2017) or aluminum (Ogunlade et al. 2020). SFN can protect against testicular apoptosis triggered by angiotensin II (Wang et al. 2017) or diabetes (Wang et al. 2014). However, it remains elusive whether SFN can ameliorate the testicular toxicity of DEHP.

The objective of this study was to explore the protective effect of SFN on the testicular toxicity of DEHP in adult rats and the underlying mechanisms. This can provide promising insights into preserving the testicular functionality and institute new solutions for the global plastic hazards.

## Materials and methods

### Drugs and chemicals

SFN was purchased from Double Wood LLC (Philadelphia, PA, USA). DEHP was provided from Loba Chemie (Colaba, Mumbai, India). All other chemicals were obtained from Sigma-Aldrich (St. Louis, MO, USA).

### Animals

Five-week-old adult male Wistar Albino rats weighing 130–140 g were obtained from the animal facility of Zagazig University. Animals were placed in plastic cages with wood chip bedding in the animal house of the Faculty of Pharmacy, Zagazig University, at a temperature of 22–24 °C, under 12-h light/dark cycle, and with free access to food and water. The animal procedures started after 1 week of acclimatization. All the guidelines of the National Institutes of Health (NIH) for animal research were strictly adopted. The animal protocol was accepted by the local ethical committee of Zagazig University (ZU/IACUC/3/F/430/2022).

### Experimental protocol and treatments

After 1 week of acclimatization, rats were randomly distributed into three groups (*n* = 6): (i) CON group, rats received matched volumes of corn oil (5 ml/kg) orally for 4 weeks; (ii) DEHP group, rats received DEHP (2 g/kg dissolved in corn oil, PO) for 4 weeks; and (iii) DEHP + SFN group, rats received SFN (10 mg/kg dissolved in distilled water, PO) for 5 weeks along with DEHP (2 g/kg dissolved in corn oil, PO) for the last 4 weeks only. The dose and duration of DEHP were selected according to prior studies (Abd El-Fattah et al. 2016; Park et al. 2002). The selected dose of DEHP has been reported to induce testicular toxicity in animal models. The dose of SFN was chosen based on a previous report (Huo et al. 2019). SFN in this dose level is considered the cutoff for minimal dose effects of SFN in preclinical in vivo studies.

Twenty-four hours after the last treatment, rats were anesthetized using 2% isoflurane. Samples of blood were withdrawn from the retro-orbital puncture and then centrifuged at 3000 rpm to get serum used for further biochemical analyses. Rats were sacrificed by cervical dislocation under anesthesia using sodium pentobarbital (40 mg/kg, IP). Testes were dissected out then weighed and expressed relative to the corresponding body weight. One testis was immediately placed in 10% formalin for histological evaluation. The other testis was snap frozen in liquid nitrogen and then stored at − 80 °C for biochemical measurement. The cauda epididymis was freshly processed for sperm analysis.

### Sperm analysis

The cauda epididymis was excised and crushed in a sterile Petri dish containing warm saline at a temperature of 37 °C. Sterilized pair of scissors was used to soften the epididymis yielding an epididymal suspension. One drop of the suspension was placed onto a warm glass slide and examined by the light microscope at power × 40. The individual motility of the sperm was examined using the light microscope, and the motility percent was evaluated by examining several microscopic fields (Galal et al. 2016).

To obtain the sperm count, 200 µl of semen was transferred by an automatic pipette into a plastic graduated tube containing a mix of 800 µl of normal saline and a few drops of 40% formalin. Then, a hemocytometer counting chamber was used to count the spermatozoa in the tube (Narayana et al. 2005). To measure the sperm deformity percentage, one drop of the semen was placed on a pre-warmed glass slide. Two drops of eosin-nigrosin stain were added to the semen. After drying, the mixture was smeared out and evaluated under an oil immersion lens. Two hundred sperm were randomly chosen in different microscopic fields and examined to evaluate the percent of abnormal spermatozoa for each rat (Elrashidy and Hasan 2020).

### Hormonal analysis

Serum concentrations of testosterone, follicular stimulating hormone (FSH), and luteinizing hormone (LH) were determined using the commercial rat ELISA kits (Bioassay, China Cat# E0182Ra, E0179Ra and E2421Ra, respectively), following the manufacturers’ instructions.

### ELISA measurements for tissue parameters

Frozen testicular tissue was homogenized in phosphate buffered saline (PBS) with a glass homogenizer on ice. Tissue homogenate was processed to measure the levels of aromatase, NRF-2, OH-1, steroidogenic acute regulatory protein (STAR), 17-beta hydroxysteroid dehydrogenase (17β-HSD), acyl Co-A synthase type 4 (ACSL4), cyclooxygenase type-2 (COX-2), and the solute carrier family 7 member 11 (SLC711A), utilizing the commercial rat ELISA kits (Bioassay, China; Cat# E0259Ra, E2184Ra, E0676Ra, E2489Ra, E1832Ra, E2932Ra, E1373Ra and E3319Ra respectively), following the manufacturers’ protocols. Expression of glutathione peroxidase-4 (GPX-4) was measured in testicular homogenate using rat ELISA kit (MyBioSource, CA, USA; Cat # MBS934198) based on the suppliers’ guidelines.

### Testicular iron measurement

Testicular tissue was washed and digested in 3:2 nitric acid/perchloric acid mixture. Iron content in testicular tissue digest was measured using atomic absorption spectrophotometer as previously described (El-Sheikh et al. 2018). Results were expressed as ppm/mg tissue weight.

### Assessment of redox status

The total antioxidant capacity (TAC), levels of malondialdehyde (MDA), and reduced glutathione (GSH) were measured in testicular tissue homogenate using the colorimetric diagnostic kits (Bio Diagnostic, Egypt; Cat# TA2513, MD2529 and GR2511, respectively) according to the suppliers’ guidelines.

### Histological and morphometric analyses

After fixation in 10% buffered formalin, the paraffin-embedded testicular tissue was de-paraffinized and cut into 5-µm-thick sections. To assess the general testicular architecture, the slices were stained with H&E stain and examined under a light microscope (Primo Star, ZEISS, China). Masson’s trichrome staining was applied to reveal the deposition of collagen fibers. However, Perls’ Prussian blue (PPB) staining was used to recognize the localization of iron within the testicular tissue. The photomicrographs were taken with Axiocam ERc 5 s camera (ZEISS, China) at the Pathology Department, Faculty of Veterinary Medicine, Zagazig University.

Five slides from five different rats per each group were chosen for morphometric analysis using ImageJ software (NIH, MD, USA), as previously reported (Karimi et al. 2018). Five random seminiferous tubules per slide were selected in H&E-stained sections to measure tubular epithelial thickness and tubular diameter. The epithelial thickness was measured from the basement membrane to the lumen. The mean diameter of the tubular cross section was determined by measuring the diameter along the major and minor axes. Round or nearly round seminiferous tubules were randomly chosen for measurement. For establishing scale and converting values from pixels to micrometers, a photograph of known distance in micrometers was employed. The percentage of fibrotic area was measured in five randomly chosen non-overlapping microscopic fields per slide in Masson’s trichrome-stained sections using Image-Pro Plus software.

### Statistical analysis

Analysis of results was processed using GraphPad Prism version 8 statistic software (GraphPad Software, Inc., La Jolla, CA, USA). To compare among different experimental groups, one-way ANOVA followed by Tukey’s post hoc test was applied. The statistical significance was assumed when *p* value was less than 0.05. All results were expressed as the mean ± standard deviation (SD).

## Results

### Effects of DEHP and SFN on body and testicular weights of rats

As illustrated in Table 1, there were no significant distinctions in the initial body weights of rats among different experimental groups. Administration of 2 g/kg/day DEHP to rats caused a significant body weight loss after 4 weeks related to CON group (*p* < 0.05). This was associated with a considerable decline of testicular weight and testicular index (*p* < 0.001) in DEHP group relative to CON group, suggesting testicular atrophy. Treatment with SFN tended to alleviate DEHP-induced testicular atrophy but did not reach statistical significance.

**Table 1:** Effects of di-2-ethylhexyl phthalate (DEHP, 2 g/kg/day, 4 weeks) and sulforaphane (SFN, 10 mg/kg/day, 5 weeks) on body and testicular weights of rats

	CON	DEHP	DEHP + SFN
Initial BW (g)	141.4 ± 15.42	140.0 ± 17.92	136.4 ± 4.89
Final BW (g)	284.6 ± 15.33	229.4 ± 47.32*	233.1 ± 55.29*
Testicular weights (mg)	3.16 ± 0.39	0.99 ± 0.21**	1.25 ± 0.24**
Testicular index*10^−3^	11.14 ± 1.49	4.51 ± 0.71**	5.36 ± 0.47**

Data are presented as mean ± SD (*n* = 6),*p < 0.05, **p < 0.001 relative to CON; *BW*, body weight; testicular index, the ratio between testicular weight and body weight

### SFN attenuates the detrimental effect of DEHP on testicular gross morphology and histological architecture in rats

**Fig. 1:**
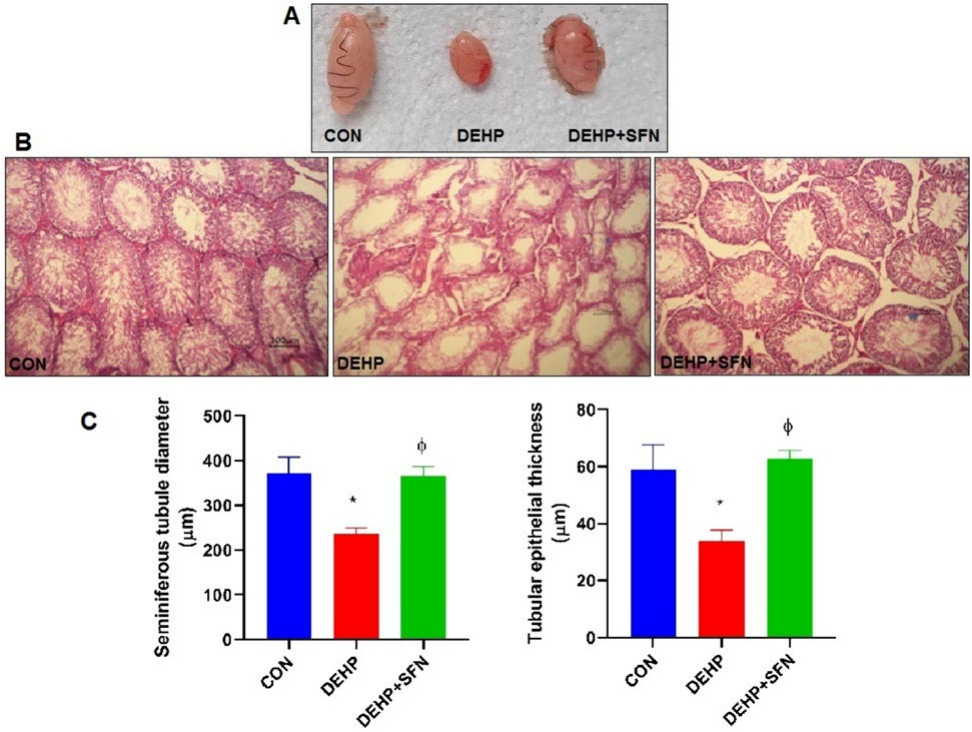
Photomicrographs of testicular gross morphology and H&E-stained testicular sections. **A** Gross morphology of testes of rats from different groups; **B** testicular sections from CON group are showing normal histological features of seminiferous tubules and interstitium. Testicular sections from DEHP group are showing distorted seminiferous tubules and arrest of spermatogenesis (arrow) with partial or complete loss of their epithelia and replaced by eosinophilic debris (star). In contrast, testicular sections from DEHP + SFN group are showing restoration of tubular morphology and epithelia (arrow) and wide interstitium (star) (H&E stain; × 200; scale bar = 100 µm). **C** Morphometric analysis of seminiferous tubular diameter and tubular epithelial thickness in H&E-stained sections; bars represent mean ± SD (*n* = 25 field/group); *p < 0.001 relative to CON group; ^ɸ^*p* < 0.001 relative to DEHP group

As presented in Fig. 1, gross morphology of testis from DEHP group indicated apparent testicular atrophy, relative to CON group. Treatment with SFN moderately improved the testicular gross morphology (Fig. 1A). On the other hand, examination of H&E-stained testicular sections from CON group revealed that all seminiferous tubules, interstitium, and tunica albuginea were apparently normal with active spermatogenesis. On the contrary, testicular sections from DEHP group showed distorted seminiferous tubules with arrest of spermatogenesis. Their lumina were empty with reduction or complete loss of their epithelia which usually suffered from apoptosis or necrosis and replaced by eosinophilic debris. Testicular sections from DEHP + SFN group furnished moderate spermatogenesis with restoring of tubular morphology and epithelial lining and wide interstitium (Fig. 1B). Morphometric analysis demonstrated that exposure of rats to DEHP caused a significant reduction of seminiferous tubular diameter and tubular epithelial thickness compared to CON group (*p* < 0.001). Supplementation of SFN significantly increased both parameters, in comparison with DEHP group (*p* < 0.001), and almost restored them to that of CON group (Fig. 1C).

### SFN attenuates testicular interstitial fibrosis in DEHP-exposed rats

**Fig. 2:**
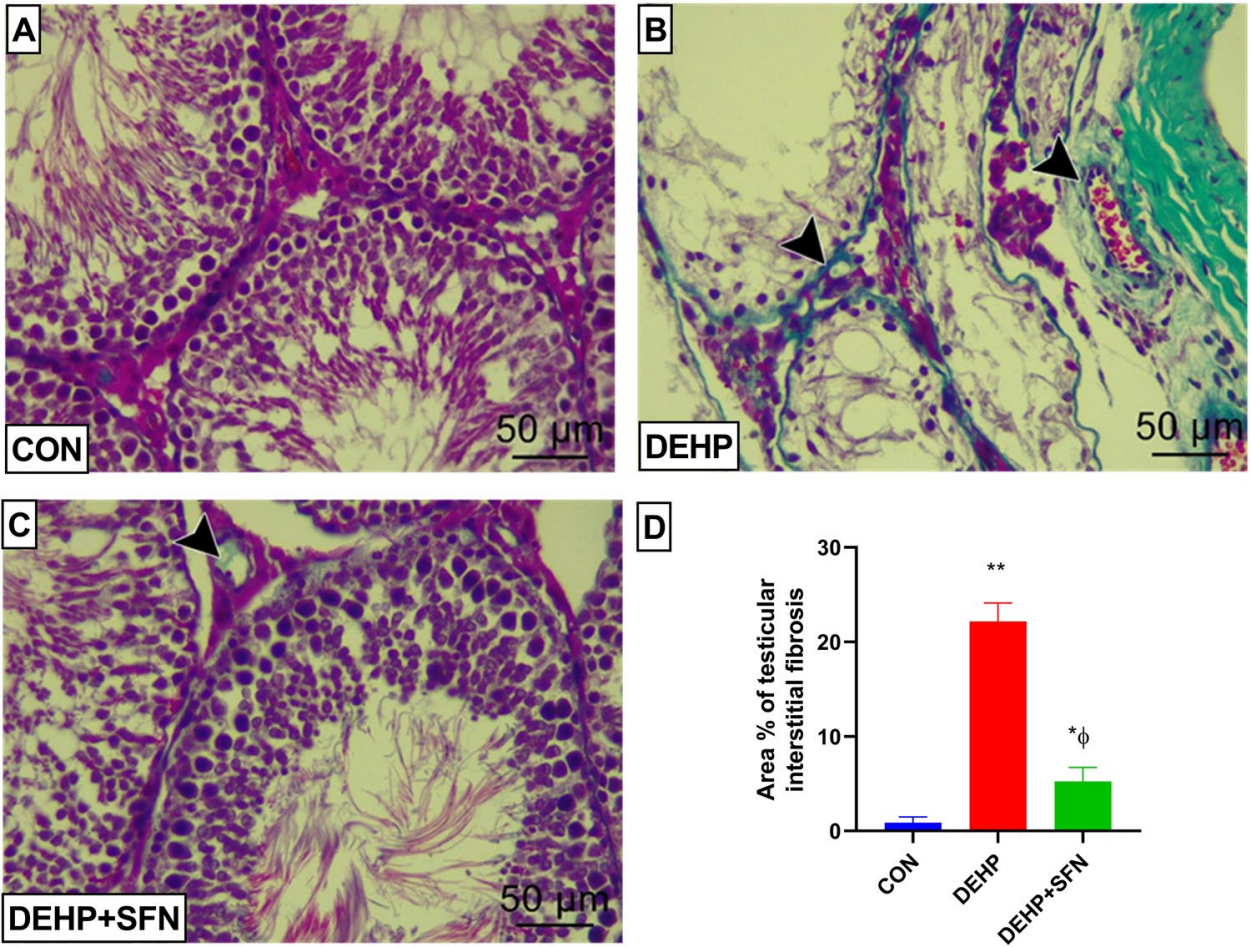
Representative photomicrographs of Masson's trichrome-stained testicular sections from experimental groups. **A** Testicular sections from CON group are showing minimal collagen fibers in basal lamina around the seminiferous tubule and walls of blood vessels in the interstitial tissue; **B** testicular sections from DEHP group are revealing massive collagen fibers (black arrow heads) surrounding the seminiferous tubules and the interstitial blood vessel; **C** testicular sections from DEHP + SFN group are revealing mild to moderate deposition of collagen fibers (black arrow head) around the seminiferous tubules and the interstitial blood vessel (Masson’s trichrome stain; × 400; scale bar = 50 µm); **D** statistical analysis of the area percentage of fibrotic tissue in testes of rats. Bars represent mean ± SD (*n* = 25 field/group); *p < 0.01, **p < 0.001 relative to CON group; ^ɸ^*p* < 0.001 relative to DEHP group

To uncover the possible fibrotic alterations in testicular tissue of rats upon DEHP exposure, testicular sections from experimental groups were stained by Masson’s trichrome stain to reveal to deposition of collagen fibers. As demonstrated in Fig. 2, examination of testicular tissue from CON group unveiled minimal deposition of collagen fibers surrounding the seminiferous tubules and walls of blood in the interstitial space (Fig. 2A). On the contrary, abundant collagen deposition was observed around both the seminiferous tubules and the interstitial blood vessel in DEHP group (Fig. 2B). However, DEHP + SFN group showed mild to moderate deposition of collagen fibers (Fig. 2C). These findings were further confirmed by the statistical analysis of the quantified results (Fig. 2D). The percentage of fibrotic area dramatically increased in testicular tissue of DEHP group, relative to CON group (*p* < 0.001). Conversely, there was a considerable reduction in the area percentage of testicular fibrosis in DEHP + SFN group, compared to DEHP group (*p* < 0.001), but still significantly higher than in the CON group (*p* < 0.01). Collectively, these findings suggested the anti-fibrotic effect of SFN.

### SFN increases the sperm count and quality in DEHP-exposed rats

**Fig. 3:**
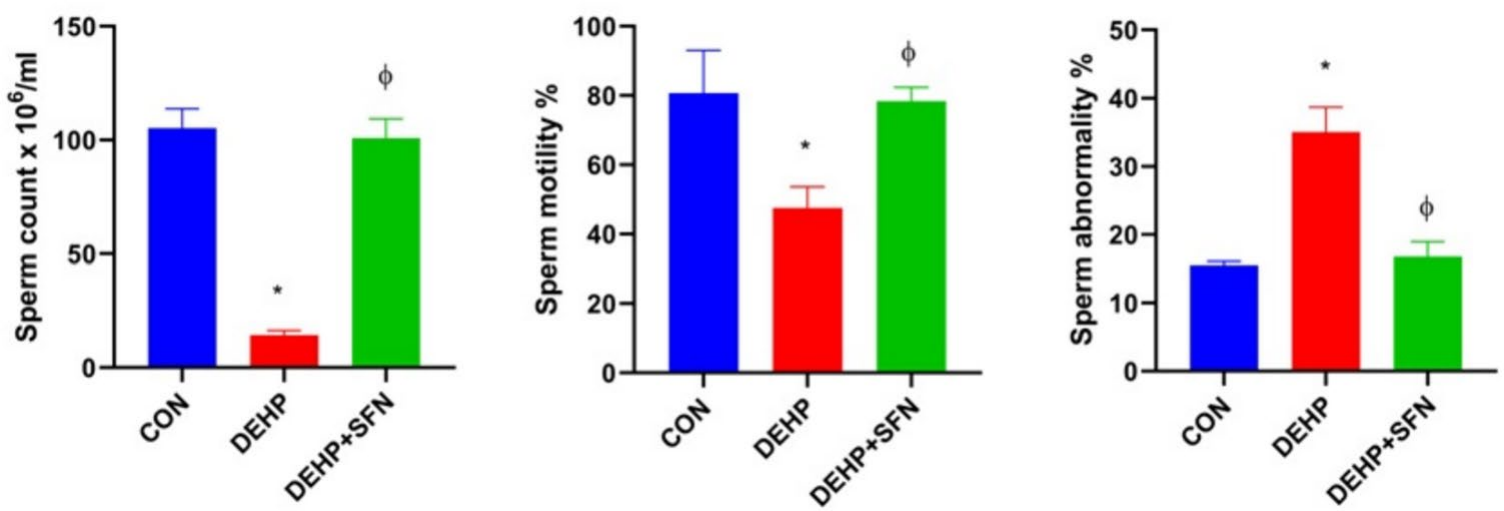
Effects of di-2-ethylhexyl phthalate (DEHP, 2 g/kg/day, 4 weeks) and sulforaphane (SFN, 10 mg/kg/day, 5 weeks) on sperm analysis. Bars represent mean ± SD (*n* = 5); *p < 0.001 relative to CON group; ^ɸ^*p* < 0.001 relative to DEHP group

As presented in Fig. 3, DEHP-exposed rats displayed marked reductions in the sperm production and motility, but elevated sperm abnormalities with respect to CON group (*p* < 0.001). Pretreatment of rats with SFN reversed the detrimental effects of DEHP on the studied sperm parameters, as compared to DEHP group (*p* < 0.001) and successfully normalized them to that of CON group.

### SFN increases serum testosterone level and promotes testicular steroidogenesis in DEHP-exposed rats

**Fig. 4:**
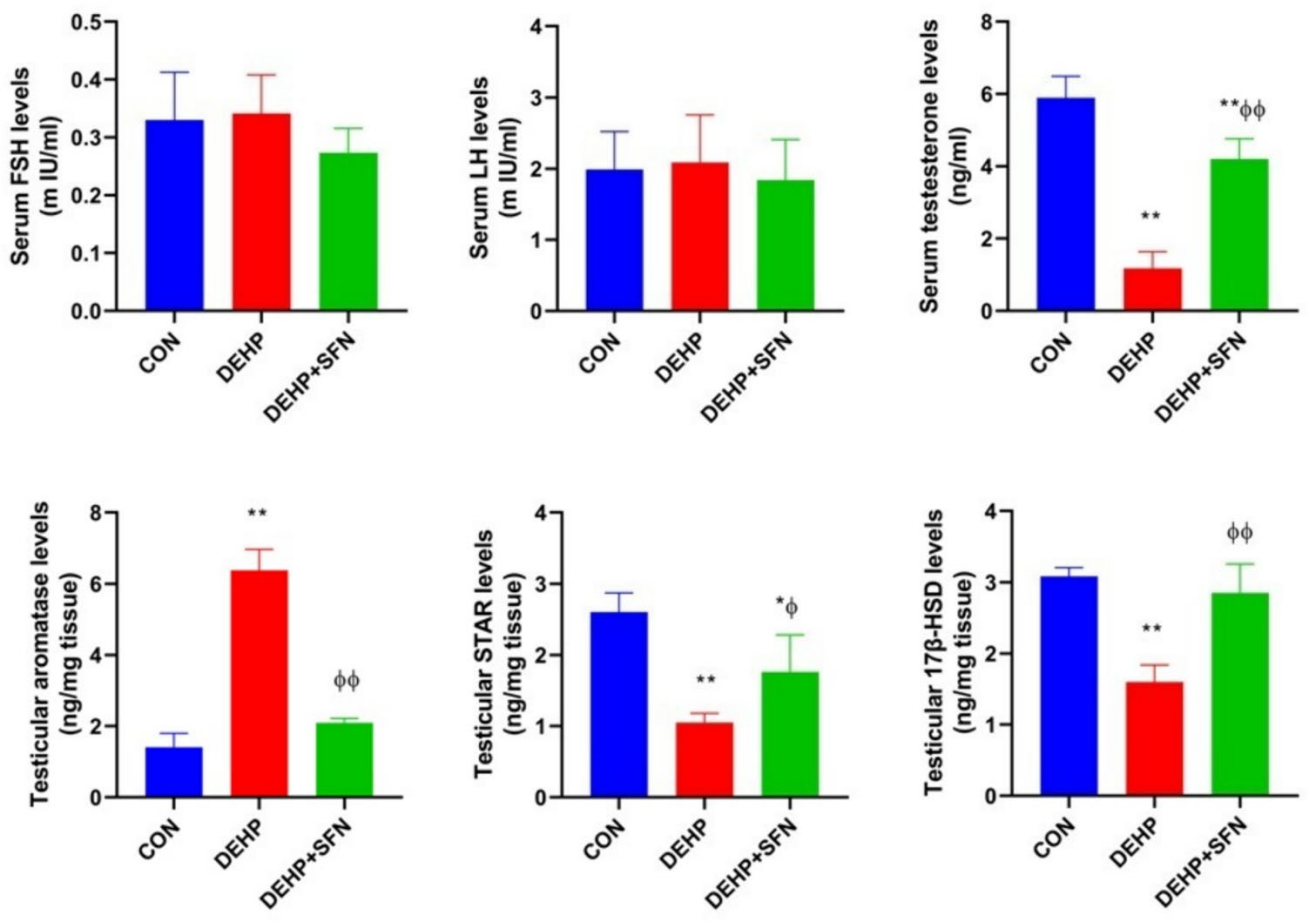
Effects of di-2-ethylhexyl phthalate (DEHP, 2 g/kg/day, 4 weeks) and sulforaphane (SFN, 10 mg/kg/day, 5 weeks) on circulating hormones and testicular aromatase and steroidogenic markers in rats. Bars represent mean ± SD (*n* = 6); *p < 0.01, **p < 0.001 relative to CON group; ^ɸ^*p* < 0.05, ^ɸɸ^*p* < 0.001 relative to DEHP group; FSH, follicular stimulating hormone; LH, luteinizing hormone; STAR, steroidogenic acute regulatory protein; 17β-HSD, 17-beta hydroxysteroid dehydrogenase

As illustrated in Fig. 4, there were no considerable distinctions in serum levels of FSH and LH among different experimental groups, which further excluded the incidence of hypothalamic-pituitary-related hypogonadism. DEHP-exposed rats displayed significantly lesser serum testosterone level than CON group (*p* < 0.001). Aromatase enzyme is responsible for the irreversible conversion of testosterone into estrogen. Compared to CON group, a significant rise in testicular aromatase level was detected in DEHP-exposed rats (*p* < 0.001). Treatment of rats with SFN concurrently with DEHP significantly increased serum testosterone level while normalized testicular aromatase level (*p* < 0.001).

To further delineate the potential effect of SFN on steroidogenesis in testicular tissue, the protein levels of STAR and 17β-HSD were evaluated, as crucial proteins in the testicular steroidogenic pathway. There was marked downregulation of the protein expression of STAR and 17β-HSD in testes of DEHP-subjected rats, as compared to CON group (*p* < 0.001), suggesting deteriorated steroidogenesis in testes of DEHP-exposed rats. Conversely, concurrent administration of SFN significantly enhanced the testicular levels of STAR (*p* < 0.05) and 17β-HSD (*p* < 0.001) relative to DEHP group. These findings underscored the beneficial effects of SFN on testicular steroidogenesis.

### SFN enhances NRF-2/HO-1 signaling and the antioxidant defense system in testes of DEHP-exposed rats

**Fig. 5:**
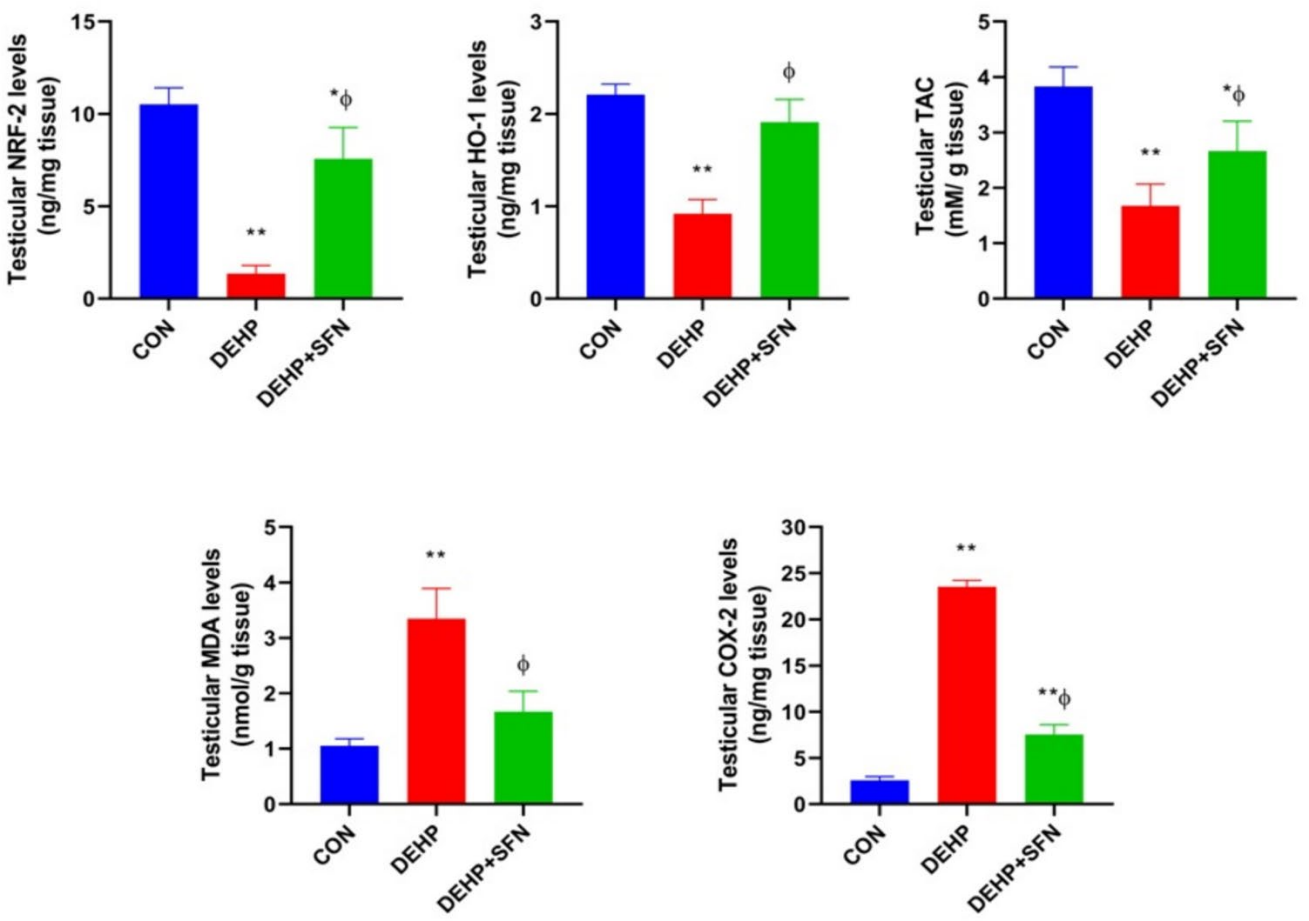
Effects of di-2-ethylhexyl phthalate (DEHP, 2 g/kg/day, 4 weeks) and sulforaphane (SFN, 10 mg/kg/day, 5 weeks) on redox status in testicular tissue of rats. Bars represent mean ± SD (*n* = 6); *p < 0.01, **p < 0.001 relative to CON group; ^ɸ^*p* < 0.001 relative to DEHP group; NRF-2, nuclear factor erythroid 2-related factor 2; HO-1, heme oxygenase-1; TAC, total antioxidant capacity; MDA, malondialdehyde; COX-2, cyclooxygenase-2

The protein expressions of NRF-2 and its downstream target HO-1 were monitored as the principal transcription factor controlling the cellular redox status and the antioxidant enzymes activity. As shown in Fig. 5, exposure of rats to DEHP markedly disrupted the testicular redox homeostasis. DEHP-subjected rats displayed a robust downregulation of NRF-2 and HO-1, coupled with a marked decline of TAC in their testicular tissue, relative to CON group (*p* < 0.001). These finding depicted the impairment of NRF-2/HO-1 system in testicular tissue of DEHP-exposed rats. Additionally there were significantly higher testicular levels of MDA and COX-2 in DEHP group than CON one (*p* < 0.001), suggesting the enhanced oxidative stress. Co-treatment with SFN enhanced NRF-2, HO-1, and TAC levels while suppressed the increased levels of MDA and COX-2 in testicular tissue of rats (*p* < 0.001). These findings collectively pointed out the activation of NRF-2/HO-1 system by SFN, which further highlighted the antioxidant properties of SFN.

### SFN counteracts testicular iron accumulation in DEHP-exposed rats

**Fig. 6:**
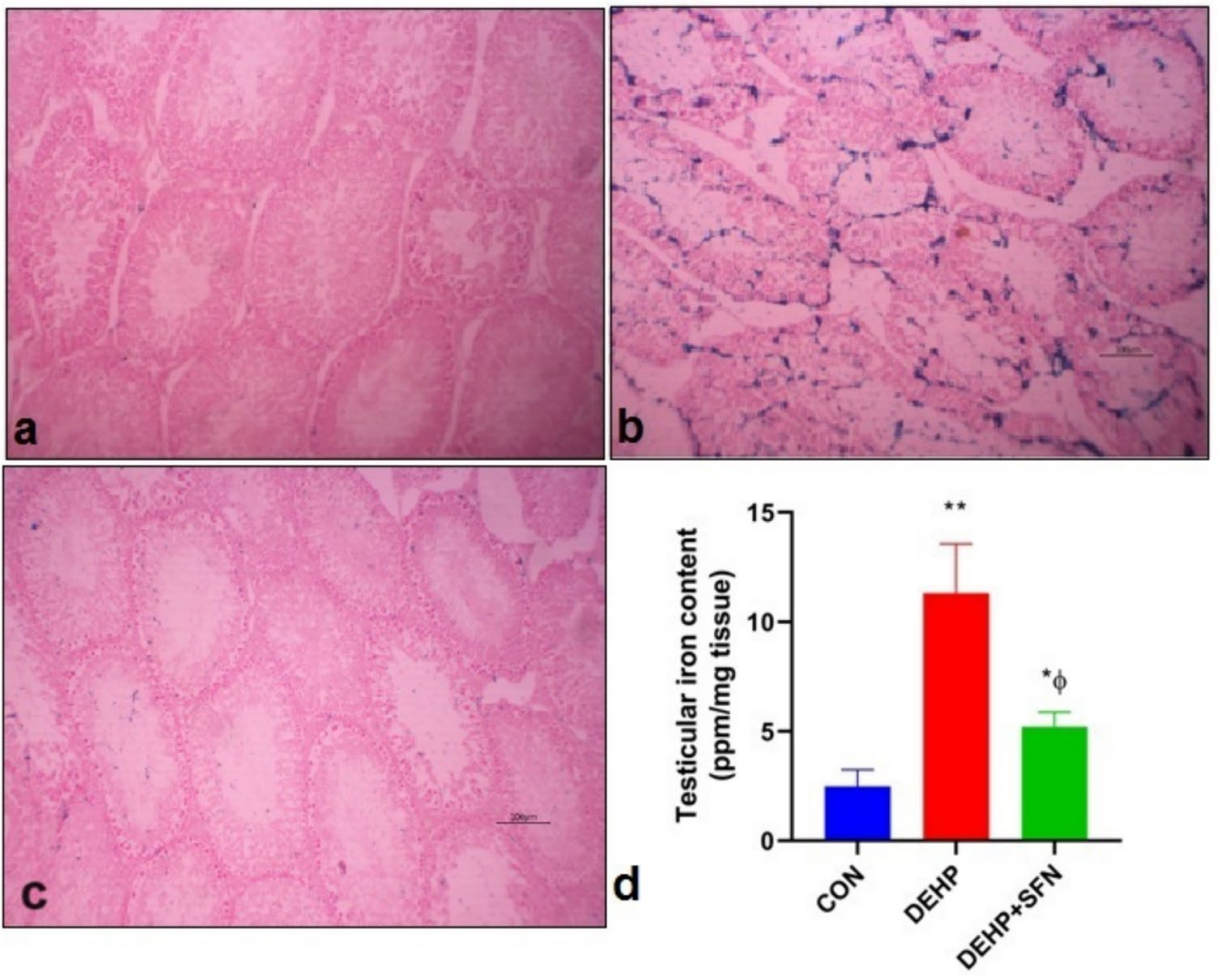
Photomicrographs of Perls’ Prussian blue (PPB)-stained testicular sections from experimental groups and quantification of iron content in testicular tissue of rats. **a** PPB-stained testicular sections from CON group showing no stain for iron; **b** PPB-stained testicular sections DEHP group showing intense stain for iron (blue color) within the seminiferous tubules; **c** PPB-stained testicular sections DEHP + SFN group showing mild stain for iron within the seminiferous tubules (PPB stain; × 200; scale bar = 100 µm); **d** Quantification of iron levels in testicular tissue of rats by atomic absorption spectroscopy; bars represent mean ± SD (*n* = 6); *p < 0.05, **p < 0.001 relative to CON group; ^ɸ^*p* < 0.001 relative to DEHP group

Iron is a crucial initiator of ferroptotic cascade. Therefore, the testicular iron content and distribution were analyzed in testicular tissue to explore the effect of DEHP on iron homeostasis. The PPB staining was conducted to illuminate the distribution of iron in different testicular compartments. As shown in Fig. 6, the results showed no PPB staining for iron in testicular sections from CON group (Fig. 6a). In contrast, testicular sections from DEHP group furnished intense positive reaction manifested as distinct blue stain within the seminiferous tubules (Fig. 6b). Meanwhile, mild stain was observed in testicular sections from DEHP + SFN group (Fig. 6c).

In order to confirm the histological findings, iron content in testicular tissue was also analyzed by atomic absorption spectroscopy (Fig. 6d). The exposure of rats to DEHP induced a significant iron accumulation in testicular tissue when compared to CON group (*p* < 0.001). Co-administration of SFN significantly lowered the iron levels in testicular tissue of rats relative to DEHP group (*p* < 0.001), but still significantly higher than CON group (*p* < 0.05). Altogether, these findings suggested that SFN mitigated the DEHP-induced testicular iron overload in rats.

### SFN upregulates SLC7A11/GPX-4 axis and offsets ferroptosis in testes of DEHP-exposed rats

**Fig. 7:**
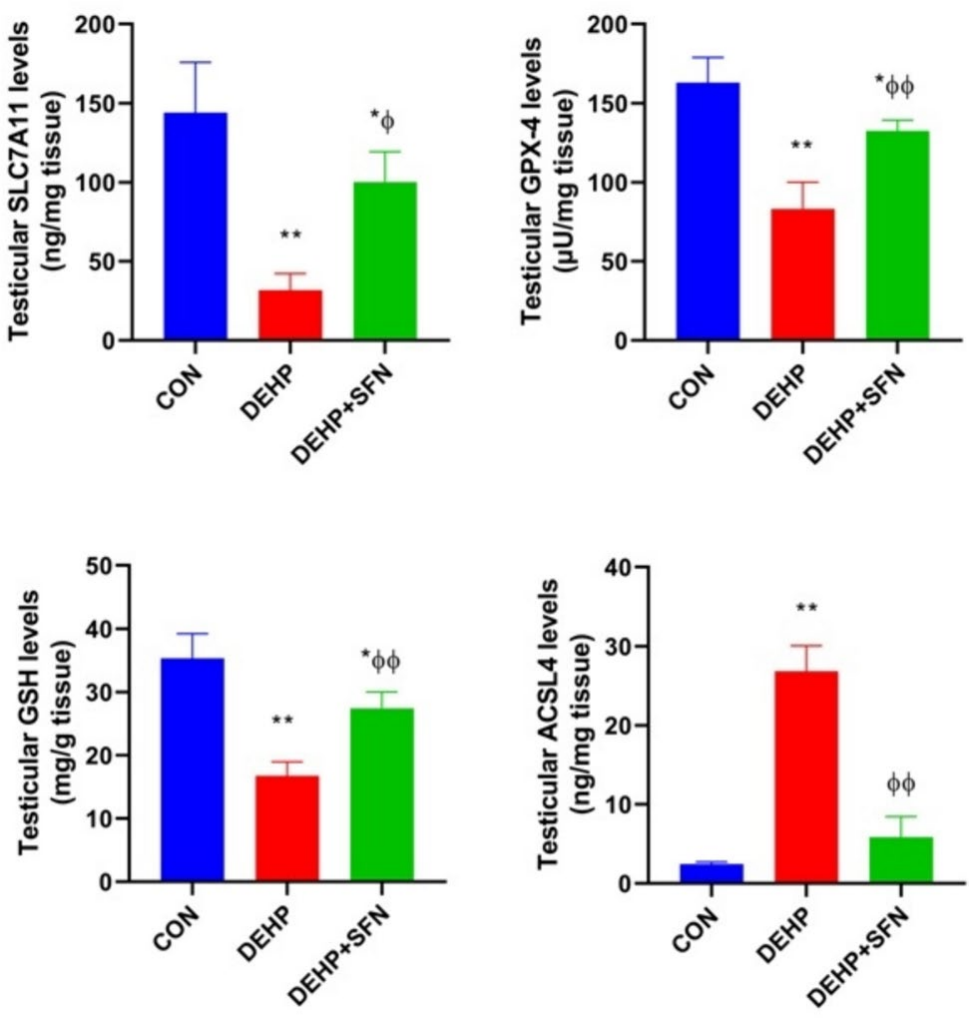
Effects of di-2-ethylhexyl phthalate (DEHP, 2 g/kg/day, 4 weeks) and sulforaphane (SFN, 10 mg/kg/day, 5 weeks) on ferroptosis markers in testicular tissue of rats. Bars represent mean ± SD (*n* = 6); *p < 0.01, **p < 0.001 relative to CON group; ^ɸ^*p* < 0.01, ^ɸɸ^*p* < 0.001 relative to DEHP group; SLC7A11, cystine/glutamate antiporter solute carrier family 7 member 11; GPX-4, glutathione peroxidae-4; GSH, reduced glutathione; ACSL4, acyl Co-A synthase type 4

In order to pursue the significance of ferroptosis in DEHP-induced testicular dysfunction and the putative ameliorative effect of SFN, the protein levels of GPX-4, SLC7A11, GSH, and ACSL4 were measured as the major regulators of ferroptosis process. It has been evident that SLC7A11, GPX-4, and GSH can negatively regulate ferroptosis. However, ACSL4 is recognized as the main inducer of ferroptotic cell death (Chen et al. 2021). As depicted in Fig. 7, DEHP-subjected rats showed marked reductions in GPX-4, SLC7A11, and GSH levels, upon comparing to CON group (*p* < 0.001). Conversely, there was a profound elevation in ACSL4 level in DEHP-exposed rats relative to CON ones (*p* < 0.001). These findings illustrated that DEHP triggered ferroptosis in testicular tissue of rats. Concurrent treatment with SFN markedly counteracted ferroptosis likely via up-surging levels of SLC7A11 (*p* < 0.01) and GSH and GPX-4 activity (*p* < 0.001) while decreasing ACSL4 levels (*p* < 0.001) when compared to DEHP group. These data uncovered the suppressive effect of SFN on ferroptosis.

## Discussion

The potential hazard of DEHP on human health is continuously increasing particularly with the prevalent use of plastic products. DEHP exposure has been linked to various toxicities in multiple body systems (Rowdhwal and Chen 2018; Zarean et al. 2016). Toxicity of the male reproductive system is a well-known effect of DEHP in humans and experimental animals. Thus, it will be of clinical importance to uncover the implicated molecular pathways and the potential protective effect of upregulating the endogenous antioxidant system by SFN.

In the present study, DEHP-exposed rats displayed apparent testicular atrophy along with deteriorated seminiferous tubules characterized by a decline of tubular diameter and epithelial thickness, loss of germ cells, and increased interstitial collagen deposition. The aberrant spermatogenesis was evident in testes of rats subjected to DEHP and largely correlated with the sperm analysis which furnished a robust drop in the sperm count and motility, along with higher sperm deformity. These findings closely agreed with prior studies (Abd-Ellah et al. 2016, Wang et al. 2021). Exposure to DEHP also caused marked body weight loss which reflected the general toxicity and the negative impact of DEHP on the overall metabolism in rats. The decline in testicular weight might be related to the small size of seminiferous tubules and/or spermatogenic arrest (Tang et al. 2018). Another plausible explanation was attributed to the enhanced apoptosis and zinc depletion in consequence to DEHP exposure (Park et al. 2002).

The toxic profile of DEHP also comprised the reduction in circulating testosterone level (Abd-Ellah et al. 2016; Tang et al. 2018). The principal site of testosterone biosynthesis is Leydig cells through steroidogenesis process. Herein, the decreased serum testosterone level might reflect Leydig cell dysfunction and impaired steroidogenesis. The downregulation of steroidogenic markers (STAR and17β-HSD) further certified this speculation. The upregulation of aromatase expression in testicular tissue of DEHP-exposed rats might also participate in reducing serum testosterone level, since testosterone was irreversibly converted into estrogen by aromatase (Choi et al. 2018). These findings exemplified the potential of DEHP to induce perturbations in the balance between androgens and estrogens. Increased aromatase expression within the testicular tissue has been shown to predispose to tumorigenesis (Fowler et al. 2000), which raised another serious concern regarding DEHP toxicity. Although DEHP is recognized as an EDC, the serum levels of gonadotropic hormones (LH & FSH) remained unchanged which might rule out the contribution of hypothalamic-pituitary gonadal axis in the observed findings in terms of diminished testosterone level. Similar results have been previously reported (Choi et al. 2018). Contradicting to our findings, a previous study has shown that exposure of adult mice to the same dose of DEHP for 2 weeks can elevate serum FSH and LH levels (Bahrami et al. 2018). The discrepancies might be owing to the differences in animal species and/or duration to DEHP exposure. Further investigations are required to monitor the temporal effects of variable doses of DEHP at several time points on the expression of gonadotropic and gonadal hormones.

Our study depicted the positive effects of SFN on testicular homeostasis. We found that SFN supplementation could ameliorate the distorted testicular architecture, reverse germ cells loss, and attenuate interstitial fibrosis in DEHP-subjected rats. Our data fit to previous findings illuminating the favorable effects of SFN on male reproductive system in different animal models (Huo et al. 2019; Ran et al. 2023). Another salient finding was the potential of SFN to restore the decline in circulatory testosterone levels associated with DEHP exposure in harmony with a published study (Jiang et al. 2017). This effect might be linked to the capability of SFN to upregulate the expression of steroidogenic proteins like STAR and 17β-HSD, which consequently promoted testosterone production. Additionally, SFN was found to effectively reverse the increased aromatase level in testes of DEHP-subjected rats, which helped to preserve the sex hormones homeostasis in testicular tissue. This might be regarded as an additional mechanism underlying SFN-induced restoration of serum testosterone levels. Given that testicular development, spermatogenesis, and male fertility are primarily regulated by testosterone (Smith and Walker 2014), therefore, it was not surprising that SFN could improve spermatogenesis in rats exposed to DEHP. This was clearly mirrored from the re-population of seminiferous tubules with germ cells as well as the escalating of sperm count and improved sperm quality in rats supplemented with SFN. Taken together, these findings highlighted SFN as a promising modality to maintain appropriate steroidogenesis and spermatogenesis which in turn preserved testicular functionality in rats.

Abundant body of evidence proposes oxidative stress as a predominant pathologic mechanism in DEHP-induced testicular toxicity (Tang et al. 2018). Consistently, we found dramatic increases in MDA and COX-2 in testes of DEHP-subjected rats which reflected the enhanced lipid peroxidation and oxidative injury. Exposure to DEHP also induced repression of NRF-2/HO-1 system which orchestrated the antioxidant defense response in testicular tissue. These findings were in agreement to previously reported studies (Abd El-Fattah et al. 2016; Zhang et al. 2018). The impaired NRF-2 signaling appeared to drive the DEHP-induced redox imbalance and decline of TAC. Interestingly, SFN supplementation likely through activation of NRF-2 could enhance TAC and offset DEHP-induced lipid peroxidation and oxidative damage in testicular tissue of rats.

One of the major consequences of oxidative stress is ferroptosis, a unique type of cell death comprised of iron-based lipid peroxidation (Chen et al. 2021). In the current research, testicular iron accumulation caused by DEHP exposure was confirmed by PPB staining and atomic absorption spectroscopy. These findings suggested that DEHP strikingly impaired testicular iron homeostasis leading to iron overload. Transferrin is a carrier protein that imports iron into the cells via transferrin receptor. Intracellular iron is stored as ferritin or undergoes ferroportin-mediated exportation out of the cell. A previous study has shown that DEHP can boost up the cellular iron uptake and storage while suppress iron exportation resulting in aberrant iron overload in spleen cells (Dai et al. 2021). Intriguingly, increased testicular iron levels have been linked to decreased testosterone synthesis and sperm dysfunction in experimental animals (Kocpinar et al. 2020; Kurniawan et al. 2019). Taken together, the aberrant iron metabolism might be another aspect of the testicular hazard of DEHP.

Lipid peroxidation is a key component in initiating ferroptosis. Intracellular iron via Fenton reaction promotes the peroxidation of membrane polyunsaturated fatty acid in the presence of ACSL4, which ultimately disrupts the membrane integrity leading to ferroptotic cell death (Yang et al. 2022b). Herein, the increased levels of ACSL4 and MDA in testes of DEHP-exposed rats furnished the enhanced testicular lipid peroxidation. This finding together with the observed iron overload further indicated that DEHP triggered ferroptosis in the testes of rats. The incidence of ferroptosis was also validated from the downregulation of SLC7A11 and GPX-4 along with marked depletion of GSH and TAC. Given that SLC7A11 and GPX-4 are considered the main repressors of ferroptotic cell death, while ACSL4 is regarded as the major driver of ferroptosis, thereby these findings unraveled that DEHP could sensitize testicular cells to initiate ferroptosis mostly via suppressing SLC7A11/GPX-4 axis while promoting ACSL4-mediated lipid peroxidation. Lately, Wang and his team have described a similar scenario in pre-pubertal mouse testes after DEHP exposure (Wang et al. 2024). Although the role of DEHP in triggering autophagy (Sun et al. 2018), apoptosis (Wang et al. 2021, Zhu et al. 2021), and pyroptosis (Hong et al. 2021) has been well-established in testicular pathologies, the contribution of ferroptosis in DEHP-induced testicular dysfunction has emerged recently. Wu et al. have shown that ferroptosis can underlie Leydig and Sertoli dysfunctions in mouse testes following DEHP exposure (Wu et al. 2022). Other researchers have highlighted ferroptosis as a key mediator in impairing the blood-testis barrier integrity and Sertoli cell homeostasis in the setting of DEHP-evoked testicular injury in mice (Yang et al. 2022a; Zhao et al. 2023).

Beyond keeping the redox homeostasis, abundant body of research data illustrate that NRF-2 can negatively regulate ferroptosis through modulating ferroptosis-related genes (Fan et al. 2017). Ablation of *Nrf-2* gene has been found to repress the protein expression of SLC7A11 and increase the accumulation of lipid peroxides (Dong et al. 2020). Additionally, NRF-2 can target genes implicated in preventing lipid peroxidation and development of the ferroptotic phenotype (Dodson et al. 2019). Therefore, pharmacological manipulation of NRF-2 signaling pathway is considered a promising intervention to combat ferroptosis-related pathologies. The current research for the first time unraveled the potential of SFN to abrogate DEHP-induced ferroptosis in testicular tissue of adult rats. SFN supplementation was found to counteract the increased testicular iron content and ACSL4, suggesting the suppressive effect of SFN on iron overload and lipid peroxidation in testicular tissue of rats. The anti-ferroptotic effect of SFN was further evidenced from the increased expression of SLC7A11 with subsequent up-surging of GPX-4 and GSH in testes of rats. These findings might explain that SFN could afford a key anti-ferroptotic function likely through activation of NRF-2/OH-1 system. In consistence with our present outcomes, a recent study has reported that SFN can alleviate hepatic ferroptosis in streptozotocin-induced diabetic mice (Savic et al. 2024). Collectively, it seemed conceivable to assume that SFN mitigated ferroptosis and rescued testicular cells which might mediate its protective effect on DEHP-induced testicular injury.

Interstitial fibrosis is the ultimate mutual path for almost all kinds of testicular injuries, regardless the initiating trigger and usually correlates with testicular dysfunction (Shiraishi et al. 2003). Herein, interstitial fibrosis was evident around seminiferous tubules in the testes of DEHP group as confirmed by Masson’s trichrome staining, which was ameliorated by SFN therapy. Given that combating fibrosis is typically associated with improvement in spermatogenesis and testicular functionality, hence, the anti-fibrotic action of SFN might be involved in its beneficial effect on testicular homeostasis. Supportively, SFN has been found to mitigate testicular fibrogenic alterations in diabetic mice (Jiang et al. 2014).

### Study limitations

The present study has some potential limitations. Firstly, we only analyzed the testicular iron level and distribution; however, the regulatory proteins which govern the iron metabolic homeostasis including import, storage, and export such as transferrin, transferrin receptor, ferritin, and ferroportin were not evaluated in the present study, which limited our ability to investigate the mechanisms behind DEHP-induced iron overload. Secondly, we investigated the effect of DEHP on lipid peroxidation in testicular tissue, but unfortunately, the expressions of 3-nitrotyrosine, protein carbonyls, 8-hydroxy-2-deoxyguanosine (8-OHdG), and γH2AX were not examined in the present study. Assessment of these oxidative stress markers will help to uncover the potential effect of DEHP on oxidative protein modification and DNA damage, respectively. Thirdly, the hypo-osmotic swelling test of spermatozoa was not conducted herein, which serves as a reliable indicator of the functional integrity of the sperm’s plasma membrane and proper male fertility. Lastly, the experimental design lacked a spermato-kinetic study which counts the individual testicular populations, namely spermatogonia, primary spermatocytes, secondary spermatocytes, spermatids, and spermatozoa, as well as somatic compartments, including Sertoli and Leydig cells. This will help to characterize the distinctive impact of DEHP pathology on the individual testicular populations. Another important issue was related to the dose of DEHP used in this study that was selected based on previous studies reported to have adverse effects on the male reproductive functions in animal models, and might be very high to be reached in the normal population. Despite these limitations, our results provide consistent data regarding the potential role of DEHP exposure in the pathogenesis of testicular dysfunction. Further investigations should consider these issues to provide a more in-depth understanding of the adverse effect of DEHP on the male reproductive system.

## Conclusion

The present research demonstrates that DEHP exposure can induce testicular atrophy and dysfunction likely through inducing oxidative injury and ferroptosis in testicular cells which ultimately deteriorates steroidogenesis and spermatogenesis in testes of male rats. These findings raise serious concerns regarding the toxic effect of DEHP on the male reproductive system that is why health professionals should counsel people to minimize the exposure to DEHP by limiting plastic products or using DEHP-free alternatives. However, these finding should be interpreted with cautions due to the differences in the toxicokinetics, and exposure dose of DEHP between humans and rodents. Our results also provide the first evidence on the potential of SFN supplementation to suppress DEHP-induced ferroptosis and testicular toxicity likely through promotion of NRF-2/ SLC7A11/GPX4 axis. These findings provide empirical basis for further clinical assessment of SFN or SFN-rich foods as promising interventions to preserve testicular function and fertility in males handling DEHP and plastic products.

## Data Availability

Data is available upon reasonable request from the corresponding author.
